# Identification and expression analysis of zebrafish *gnaq* in the hypothalamic–Pituitary–Gonadal axis

**DOI:** 10.3389/fgene.2022.1015796

**Published:** 2022-11-10

**Authors:** Chong Wang, Le Yang, Tiaoyi Xiao, Junhua Li, Qiaolin Liu, Shuting Xiong

**Affiliations:** ^1^ College of Animal Science and Technology, Hunan Agricultural University, Changsha, China; ^2^ Hunan Engineering Technology Research Center of Featured Aquatic Resources Utilization, Hunan Agricultural University, Changsha, China

**Keywords:** gnaq/Gαq, embryonic development, gonads, reproduction cycle, zebrafish

## Abstract

The G proteins have emerged as essential molecular switches in a wide variety of signal transduction pathways. Gαq, encoded by G protein subunit alpha q (*gnaq*), is a member of the G proteins and participates in regulating important biological activities in mammals; however, its function and regulatory mechanism in teleost remain largely unclear. In the current study, we cloned the cDNA of *gnaq* from zebrafish (*Danio rerio*) and investigated the expression characteristics of Gαq/*gnaq* in reproductive tissues. RT-PCR and WISH analyses showed that *gnaq* was widely expressed in zebrafish tissues, with high expression in the brain, olfactory brain, and hypothalamus. During the embryonic development stage*,* the g*naq* was mainly distributed in the hypothalamus after 72 h post-fertilization. In addition, immunohistochemistry analysis revealed that the Gαq protein was highly expressed in the diffuse nucleus of the inferior hypothalamic lobe (DIL), ventral zone of the periventricular hypothalamus (Hv), and caudal zone of the periventricular hypothalamus (Hc) in adult zebrafish. Furthermore, in the gonads, the Gαq protein was found in oocytes of all stages, except spermatids. Lastly, the *gnaq* mRNA exhibited relatively low expression in gonads on Day 4 during the reproductive cycle, while increasing drastically in the hypothalamus and pituitary afterward. Altogether, our results suggest that *gnaq*/Gαq might be important in fish reproduction.

## Introduction

The G proteins are signal transduction proteins that transduce more than 800 G protein-coupled receptors (GPCRs) into cellular responses (Hauser et al., 2018). The signal transmission from GPCRs to downstream depends on the Gα subunit. According to the Gα subunit type (Anantharaman et al., 2011), the G proteins could be divided into four functional subfamilies: Gαs (Gαs and Gαolf), Gαi/o (Gαi1, Gαi2, Gαi3, Gαo, Gαz, Gαt1, Gαt2, and Gαt3), Gαq/11 (Gαq, Gα11, Gα14, and Gα15), and Gα12/13 (Gα12 and Gα13). These Gα subunits could regulate different signal pathways (Neves et al., 2002).

Reproductive traits not only are important in directly affecting the economic output but are also essential for species continuation; reproduction is thus a critical process throughout the life cycle (Blanco, 2020). The reproduction process consists of the maturity of the sex organs and the coordination of the sex system with the nervous system (Sakai et al., 2020; Barber et al., 2021). The hypothalamic–pituitary–gonad (HPG) axis has a critical function in the reproductive process (Li X. et al., 2022; Wickramasuriya et al., 2022). Gαq, encoded by the *gnaq* gene, participates in the regulation of a wide variety of biological processes *via* pathways which include GnRH signals, oxytocin signals, and estrogen signals. *GNAQ* has been detected with high expression in the reproductive system of humans and rats (Paulssen et al., 1991; Haugen et al., 1993; Chen et al., 1996). In addition, *GNAQ* is also highly expressed on the HPG axis of Kazakh sheep, and *GNAQ* knockdown in hypothalamic nerve cells was reported to negatively regulate kisspeptin and promote GnRH gene expression through the kisspeptin-GPR54 signaling pathway (Zhu et al., 2022a). *GNAQ* (A191G) is also a potential molecular marker for controlling the seasonal reproduction and litter size of sheep (Zhu et al., 2022b), while Gαq/11 was confirmed to regulate reproductive maturation (Babwah et al., 2015). In addition, *GNAQ* knockout in mouse granulosa cells did not affect follicular development, oocyte maturation, cumulus expansion, fertilization, luteinization, or uterine receptivity, but did affect follicular rupture, thus preventing the release of oocytes (Breen et al., 2013). In conclusion, the critical role of Gαq/*GNAQ* in the GnRH pathway, HPG axis signaling, and reproduction has been widely studied in mammals, while the function of *gnaq* involving fish reproduction remains mostly a mystery.

In this study, we cloned the *gnaq* cDNA from zebrafish and analyzed its expression pattern in embryo development and reproduction. Our work provides preliminary evidence for understanding the role of *gnaq*/Gαq in the reproductive system in teleosts.

## Materials and methods

### Fish care

AB strain zebrafish were used as the experimental animal; relevant animal experimental operations were reviewed and approved by the institutional animal care and use committee (IACUC) of Hunan Agricultural University. As described previously (Xiong et al., 2018), experimental fish are raised in a 3 L or 10 L container in a recirculating aquaculture system at 28°C on a 14 h light/10 h dark cycle and are maintained in accordance with the *Guide for the Care and Use of Laboratory Animals*. Experimental zebrafish are anesthetized using tricaine methanesulfonate (MS-222) before sampling. Embryos are raised at 28°C in a constant temperature light incubator.

### Sample collection and total RNA extraction

Samples of the brain, olfactory brain, hypothalamus, pituitary, heart, liver, spleen, kidney, ovary, and testis were obtained from five female and five male zebrafish, which were collected for cloning and *gnaq* mRNA profile. Embryos in different stages (Kimmel et al., 1995) were collected for real-time fluorescent quantitative PCR and whole mount *in situ* hybridization (WISH). The five males and five females in the reproduction cycle were anesthetized, and the hypothalamus, pituitary, ovary, and testis were sampled to detect the *gnaq* mRNA expression characteristics. All these samples were immediately snap-frozen in liquid nitrogen and stored at −80°C for RNA isolation. Total RNA was extracted according to the instructions of the RNA-easy Isolation Reagent (Vazyme, China) and the cDNA was synthesized using Revert Aid™ First Strand cDNA Synthesis Kit (Thermo Fisher Scientific, United States).

### cDNA cloning, sequence analysis, and phylogenetic analysis

All primers used in our work were designed by Primer 5.0 software, synthesized by Tsingke Biotechnology Co., Ltd., and listed in Supplementary Table S1. The sequence of *gnaq* was amplificated by the transStart® FastPfu fly DNA Polymerase kit (TransGen, China). The reaction program was set as follows: 95°C for 2 min; 40 circles of 95°C for 20 s, 55°C for 20 s, 72°C for 20 s; 72°C for 5 min. PCR products were purified with the FastPure Gel DNA Extraction Mini Kit (Vazyme, China) and sequenced by Tsingke Biotechnology Co., Ltd. The conservative domain and binding sites of the Gαq protein were analyzed on NCBI (https://www.ncbi.nlm.nih.gov/Structure/cdd/wrpsb.cgi). The tertiary structure of zebrafish Gαq protein was predicted by cognate modeling on the SEISS-MODEL website (https://swissmodel.expasy.org/) referring to the structure of human Gαq protein. The amino acid sequence was aligned by DNAMAN 6.0 software. A phylogenetic tree was constructed using the maximum likelihood method and MEGA 11 software based on Gαq protein sequences from different species. A bootstrapping test was adopted with 1,000 replications and the phylogenetic tree was then edited online using the EvolView tool (https://www.evolgenius.info/evolview-v2/#login).

### qPCR and data analysis

Primer availability was confirmed by qPCR testing and the sequences were also confirmed by sequencing. Real-time fluorescent quantitative PCR was performed on a Bio-Rad PCR system by CFX96 Optics Module with chamq universal SYBR qPCR Master Mix (Vazyme, China) as described previously (Xiong et al., 2018). Each experiment was replicated three times and the data were analyzed using the 2^−ΔΔCt^ method; the abundance of mRNA was normalized to that of *β-actin* and *ef1α*. Statistical Package for Social Sciences (SPSS, version 25) software was used to analyze the statistical data of one-way ANOVA. A probability (P) of *p* < 0.05*, *p* < 0.01*** was considered statistically significant. The data were shown as mean value ±standard error; GraphPad Prism 7 software was used for mapping.

### Whole mount *in situ* hybridization

Embryos at different stages were collected and fixed with 4% paraformaldehyde (DEPC water dissolution). The coding sequence of *gnaq* was amplified and then used as the template for the preparation of probes. These were synthesized using DIG labeling mix and T7 polymerase (Roche, United States); the hybridization was conducted as previously described (Thisse and Thisse, 2008). Photographs were taken using Leica MZ16FA Microscope by ACImage software.

### Histological analysis

The ovaries and testes dissected from zebrafish during the reproductive cycle were fixed in 4% paraformaldehyde (PFA) overnight at 4°C. For histological analysis, sections were cut 5 μm and stained with hematoxylin and eosin. HE-staining was performed as described previously (Xiong et al., 2020). The stained sections were imaged and photographed by microscope (Leica, German).

### Immunohistochemistry

The testis, ovary, and hypothalamus of zebrafish were fixed with 4% paraformaldehyde for 24 h at 4°C, dehydrated in graded alcohols, cleared in toluene, and embedded in paraffin wax. Serial transverse sections 5 µm thick were dried at 60°C for 2 h and placed on glycerin/albumin-coated slides. Dewaxed sections were rehydrated and washed in phosphate buffered solution (PBS), then incubated with PBS containing 3% hydrogen peroxide for 30 min to quench endogenous peroxidase activity. Next, the sections were incubated with Gαq antibody (ABclonal, China) overnight at 4°C. For the negative control, normal pre-immune rabbit serum was used to replace the Gαq antibody. The sections were then washed thrice with PBST and incubated with goat-anti-rat IgG (Abcam, Britain 1:5,000) at 37°C for 45 min. Following this, DAB chromogenic solution (Maxim, China) was used for 3–10 min, and hematoxylin (BaSO, China) was applied for 1 min to stain the nucleus. The sections were photographed by an Olympus microscope (Olympus, Japan). The protein sample of the zebrafish brain tissue was prepared as described before (Xiong et al., 2017). Western blot assay was then applied to verify the specificity of the commercial Gαq antibody. The detailed WB protocol was referred to in a previous study (Xiong et al., 2017).

### Preparation of recombinant protein

The PCR product of zebrafish *gnaq* (Dr.*gnaq*) was integrated into the pGEX-4T-1 expression vector. The recombinant plasmid was transformed into *E. coli* BL21. IPTG was then added to the LB medium at a final concentration of 1 mM and incubated at 16°C with shaking at 180 rpm for 16 h. After induction, the bacteria were sonicated to lyse them and the supernatant was harvested. An Ni-NTA sepharose column was employed to purify recombinant GST-tagged Dr.*gnaq* proteins (rDr.Gαq). The purified recombinant proteins were dialyzed for three times against tris-buffered saline (TBS; 50 mM Tris-HCl, 150 mM NaCl, pH 7.4) at 4°C; the concentration was determined according to a BCA assay kit (Sigma-Aldrich, United States).

### Western blot

Protein extracts were sampled from the zebrafish hypothalamus and separated by 10% SDS-PAGE (ACE Biotechnology, China) and transferred onto a polyvinylidene fluoride (PVDF) membrane (Millipore, United States). The membranes were blocked with QuickBlock™ Blocking Buffer (Beyotime, China) at room temperature, and incubated with following the primary Abs overnight at 4°C: rabbit polyclonal anti-Gαq (ABclonal, China) and rabbit polyclonal anti-β-actin (HUABIO, China). The blots were detected with HRP-conjugated anti-rabbit IgG (1:2000) and visualized by an enhanced chemiluminescence (ECL) reagent (Beyotime, China).

## Results

### Characteristics of zebrafish *gnaq* sequence

**FIGURE 1:**
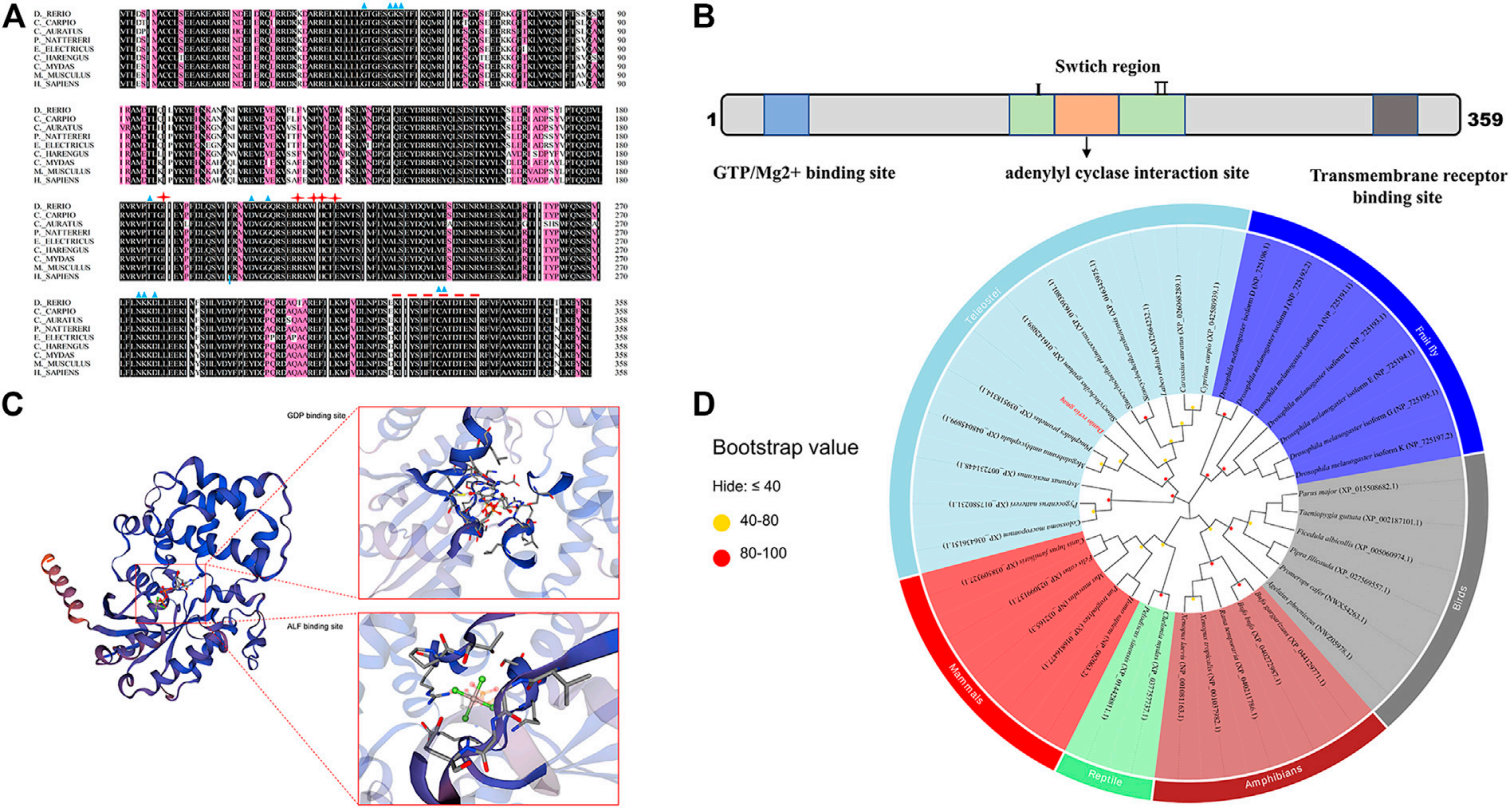
Sequence and phylogenetic analysis of *gnaq*. **(A)** Multiple sequence alignment of Gαq amino acids between zebrafish and other species. The blue triangle indicates GTP/Mg2+ binding site, red stars represent the conserved adenylyl cyclase interaction site, and the red dotted line represents transmembrane receptor binding site. Species involved in sequence alignment: (*Cyprinus carpio*, XP_042580939.1), (*Carassius auratus*, enscal00000064064), (*Pygocentrus nattereri*, XP_017580231.1), (*Electrophorus electricu*s, XP_026881183.1), (*Clupea harengus*, XP_031433518.1), (*Chelonia mydas*, XP_037757737.1), (*Mus musculus*, NP_032165.3), (*Homo sapiens*, NP_002063.2). **(B)** Linear representation of Gαq sequence motifs and binding regions. **(C)** Deduced tertiary-dimensional structure of Gαq. The structure showed the GDP binding site (T.47, E.49, S.50, G.51, K.52, S.53, T.54, S.156, L.180, R.181, V.182, R.183, V.184, N.274, K.275, D.277, L.278, C.330, A.331, T.332) and ALF binding site (G.48, E.49, K.52, S.53, R.183, P.185, T.186, V.206, G.207, G.208, Q.209). **(D)** Phylogenetic tree based on the amino acid sequences of the known Gαq proteins from various organisms. The dot at each branch represents the bootstrap values obtained with 1,000 replicates.

Zebrafish *gnaq* gene coding sequence (CDS) has 1,080 base pairs, encodes 359 amino acids, mainly has a GTP/Mg2+ binding site, a conserved adenylyl cyclase interaction site, and transmembrane receptor binding site (Figure 1A). Multiple sequence alignment showed that zebrafish Gαq has high homology with humans (92.76%, *Homo sapiens*, NP_002063.2), mice (93.04%, *Mus musculus*, NP_032165.3), common carp (95.82%, *Cyprinus carpio*, XP_042580939.1), and crucian carp (91.09%, *Carassius auratus*, enscal00000064064) (Figure 1A). Gαq consists of a GTP/Mg2+ binding site, a conserved adenylyl cyclase interaction site, and a transmembrane receptor binding site (Figure 1B). There are also two switch regions, similar to that of RGS proteins (Tesmer et al., 2005); these regions are responsible for important conformational changes between the GDP/GTP-bound forms of the protein (Kamato et al., 2017). The deduced tertiary-dimensional structure showed that Gαq has a GDP binding site (T.47, E.49, S.50, G.51, K.52, S.53, T.54, S.156, L.180, R.181, V.182, R.183, V.184, N.274, K.275, D.277, L.278, C.330, A.331, T.332) and an ALF binding site (G.48, E.49, K.52, S.53, R.183, P.185, T.186, V.206, G.207, G.208, Q.209) (Figure 1C).

Phylogenetic analysis was conducted to investigate the evolutionary relationship of *gnaq*. The maximum-likelihood method was used to construct the phylogenetic tree based on Gαq amino acid sequences from various vertebrates (mammals, birds, teleosts, reptiles, and amphibians) and invertebrates. As shown in Figure 1D, Gαq was clustered into three main branches: invertebrates, teleostei, and other vertebrates. In other vertebrate branches, mammal and reptile Gαq clustered into one clade, while bird and amphibian Gαq clustered into another. In the teleostei branch, cyprinid and non-cyprinid fish Gαq were clustered into two clades. The zebrafish Gαq and other cyprinids—such as common carp (*C. carpio*, XP_042580939.1) and crucian carp (*C. auratus,* XP_026068289.1)—gathered into a large cluster (Figure 1D). Among them, the zebrafish Gαq exhibited the highest homology with *Megalobrama amblycephala* (XP.007231448.1) and *Prmephales promelas* (XP.039518314.1) (Figure 1D).

### Tissue distribution of *gnaq* mRNA

**FIGURE 2:**
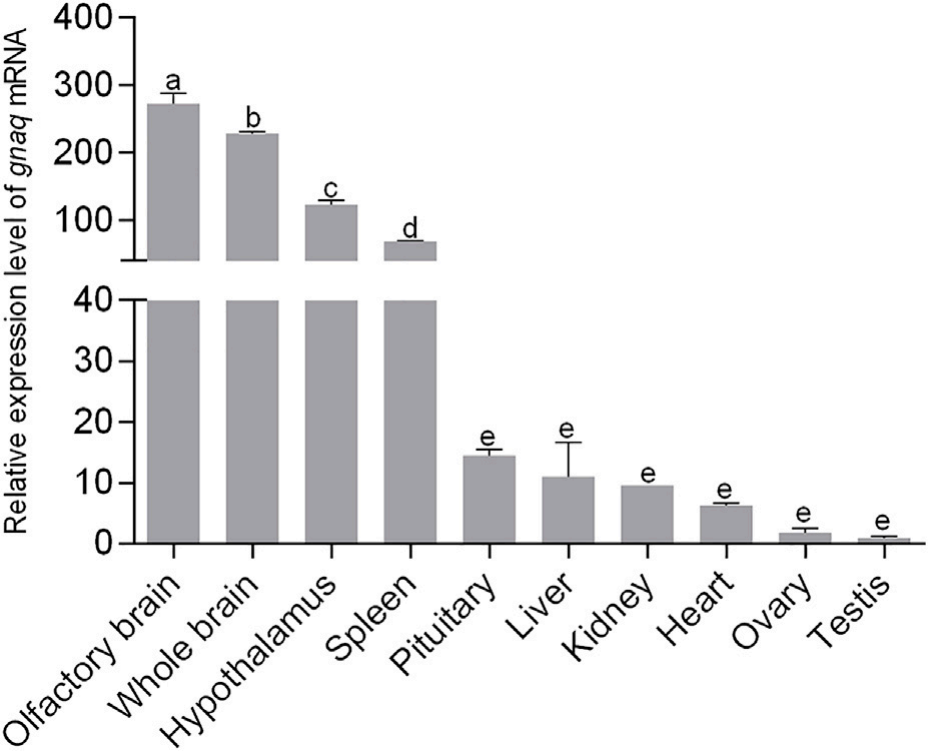
Tissue distribution of *gnaq* mRNA. Error bars indicate mean ± SEM (*n* = 3); one-way ANOVA was used for statistical analysis (*p* < 0.05).

We performed qPCR analysis to explore the expression profiles of *ganq* in different tissues of adult zebrafish. As shown in Figure 2, *gnaq* mRNA was widely expressed in the whole brain, olfactory brain, hypothalamus, pituitary, heart, liver, spleen, kidney, ovary, and testis. In addition, *gnaq* mRNA had relatively high expression in the olfactory brain, brain, hypothalamus, spleen, and pituitary, and low expression in the ovary and testis (Figure 2).

### Localization of *gnaq* mRNA during development

**FIGURE 3:**
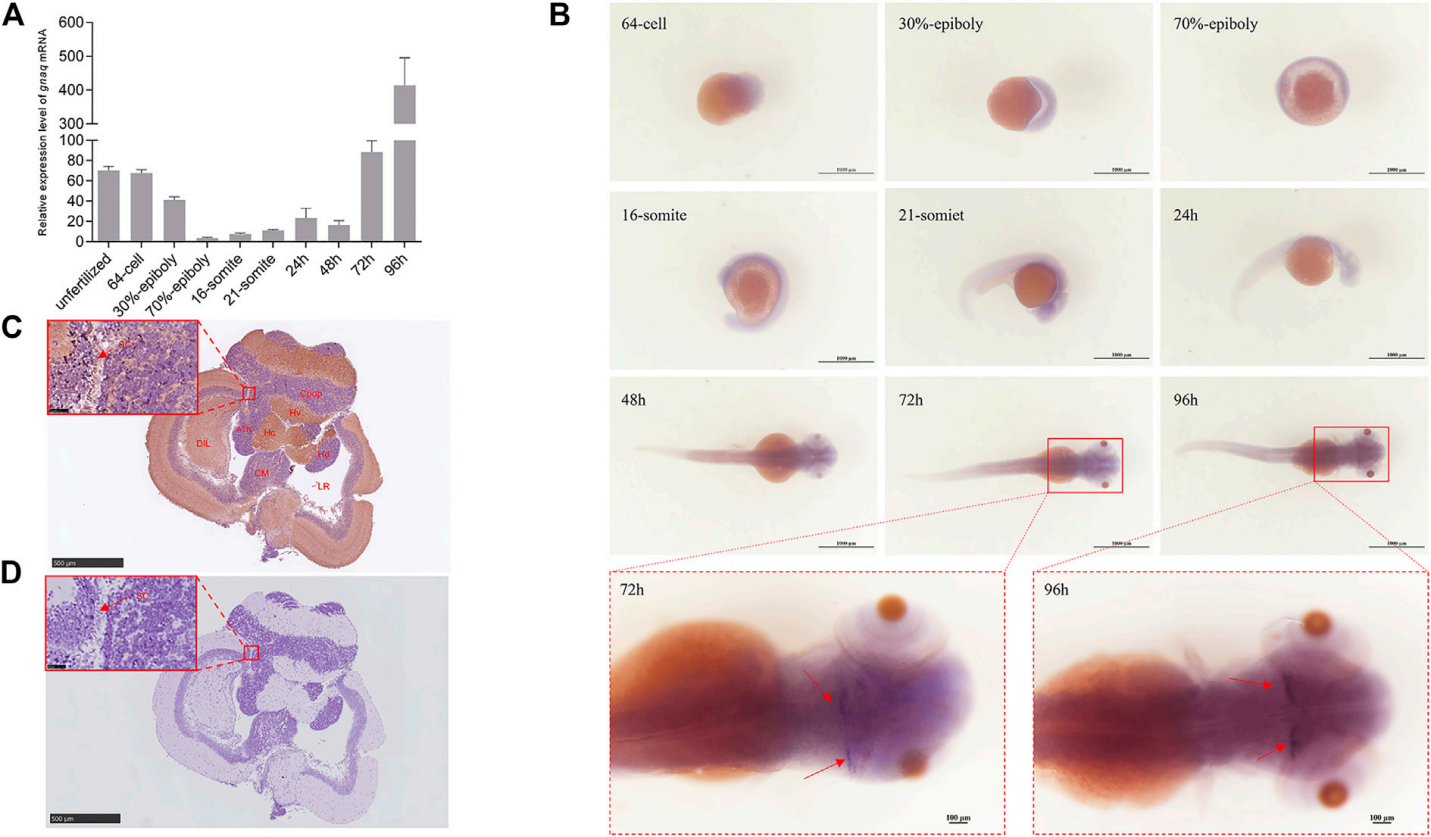
Expression and distribution of gnaq mRNA during embryonic development and Gαq in section of hypothalamus. **(A)** Relative expression of *gnaq* mRNA in the embryonic development stage; Error bars indicate mean ± SEM, *n* = 3; one-way ANOVA was used for statistical analysis (*p* < 0.05). **(B)** whole-mount *in situ* hybridizations of *gnaq*; stage: 64-cell, 30%-epiboly, 70%**-**epiboly, 16-somite, 21-somite, 24 h, 48 h, 72 h, and 96 h. Scale bar: 1000 μm (*n* = 10); **(C)** immunohistochemical analysis of Gαq in hypothalamus; SC: secretory cell; DIL: diffuse nucleus of the inferior hypothalamic lobe; ATN: anterior tuberal nucleus; Hc: caudal zone of periventricular hypothalamus; Hv: ventral zone of periventricular hypothalamus; Hd: dorsal zone of periventricular hypothalamus; Cpop: postoptic commissure; CM: corpus mammilare: LR: lateral recess; scale bar: 500 μm; **(D)** negative control; SC: secretory cell; scale bar: 500 μm.

We then studied the *gnaq* spatiotemporal expression pattern during the zebrafish embryonic development process. As revealed by qPCR (Figure 3A), the expression pattern of *gnaq* exhibits a U-shaped trend throughout the embryonic development process: *gnaq* had a high expression in unfertilized eggs and gradually decreased to an extremely weak level at 75% epiboly, soon started to increase, and peaked at 96 h post fertilization (hpf).

To further locate the *gnaq* mRNA in embryos, a whole-mount *in situ* hybridization (WISH) was applied. As seen from Figure 3B, *gnaq* was ubiquitously expressed before 90% epiboly, mainly concentrated in the animal pole, and then the *gnaq* signal gradually focused on the head after the 16-somites stage. After 72 hpf, *gnaq* was significantly expressed in the hypothalamus, and the signal was deeper at 96 hpf (Figure 3B). In addition, the sense probe was applied for the negative control: there was no positive signal in the embryo during the its development (Supplementary Figure S1).

### Location of Gαq in the hypothalamus and gonads

We continued to seek the specific location of *gnaq* in the hypothalamus. The hypothalamus sections used in this immunohistochemical experiment were sampled from adult zebrafish. The immunohistochemical results (Figure 3C) showed that Gαq had a strong signal in the diffuse nucleus of the inferior hypothalamic lobe (DIL), ventral zone of periventricular hypothalamus (Hv), and caudal zone of the periventricular hypothalamus (Hc). Weak signals were also detected in the anterior tuberal nucleus (ATN), corpus mammilare (CM), postoptic commissure (Cpop), and in the dorsal zone of the periventricular hypothalamus (Hd). No positive signals were observed in the negative control (Figure 3D).

**FIGURE 4:**
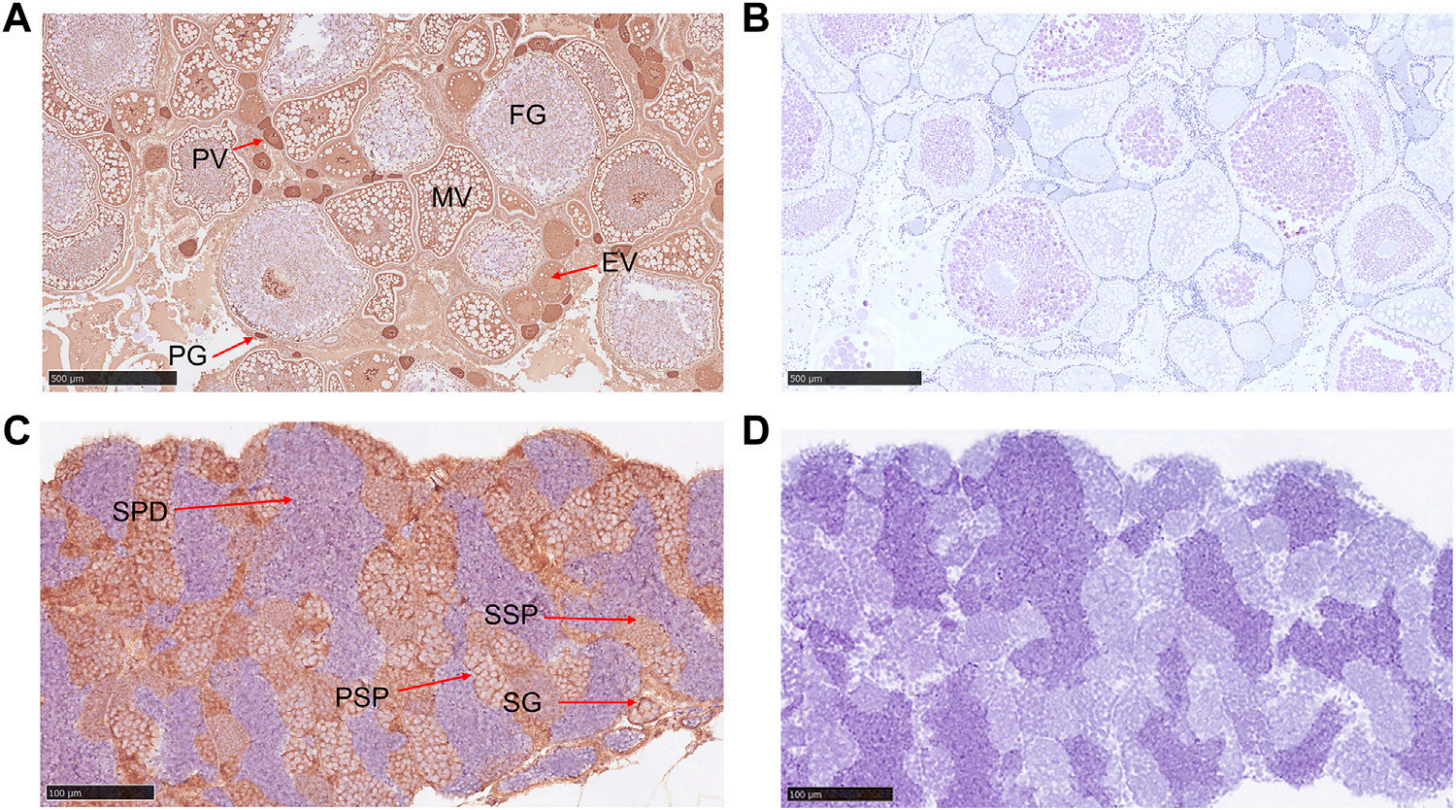
Immunohistochemical analysis of Gαq in ovary and testis. **(A)** Immunohistochemical analysis of Gαq protein in the ovary. PG: primary growth stage; PV: previtellogenic stage; EV: early vitellogenic stage; MV: midvitellogenic stage; FG: full grown stage; scale bar: 500 μm; **(B)** negative control; scale bar: 500 μm. **(C)** Immunohistochemical analysis of Gαq protein in the testis. SP: spermatogonia; PSP: primary spermatocyte; SSP: secondary spermatocyte; SPD: spermatids; scale bar: 100 μm. **(D)** Negative control; scale bar: 100 μm.

Low expression *Gnaq* mRNA was detected in the gonads; we wonder if the Gαq protein is highly expressed in gonads. Subsequently, immunohistochemical experimental results showed that the Gαq protein was present in the ovary and testis. In the ovary, Gαq immunoreactivity was detected in the oocytes of all stages, with higher expression in immature oocytes, including primary growth oocytes and previtellogenic oocytes, and lower expression in full-growth oocytes (Figure 4A). In the testis, positive signals were also observed in spermatogonia, primary spermatocytes, and secondary spermatocytes. Surprisingly, the Gαq signal was not found in spermatids (Figure 4C). No positive signals were observed in the negative control (Figures 4B,D).

The rDr.Gαq protein and the western blot assay were applied to verify the specificity of the commercial Gαq antibody. As shown in Supplementary Figure S2, the commercial Gαq antibody specifically recognizes the Gαq protein in zebrafish.

### Dynamic change of *gnaq* during the reproductive cycle

**FIGURE 5:**
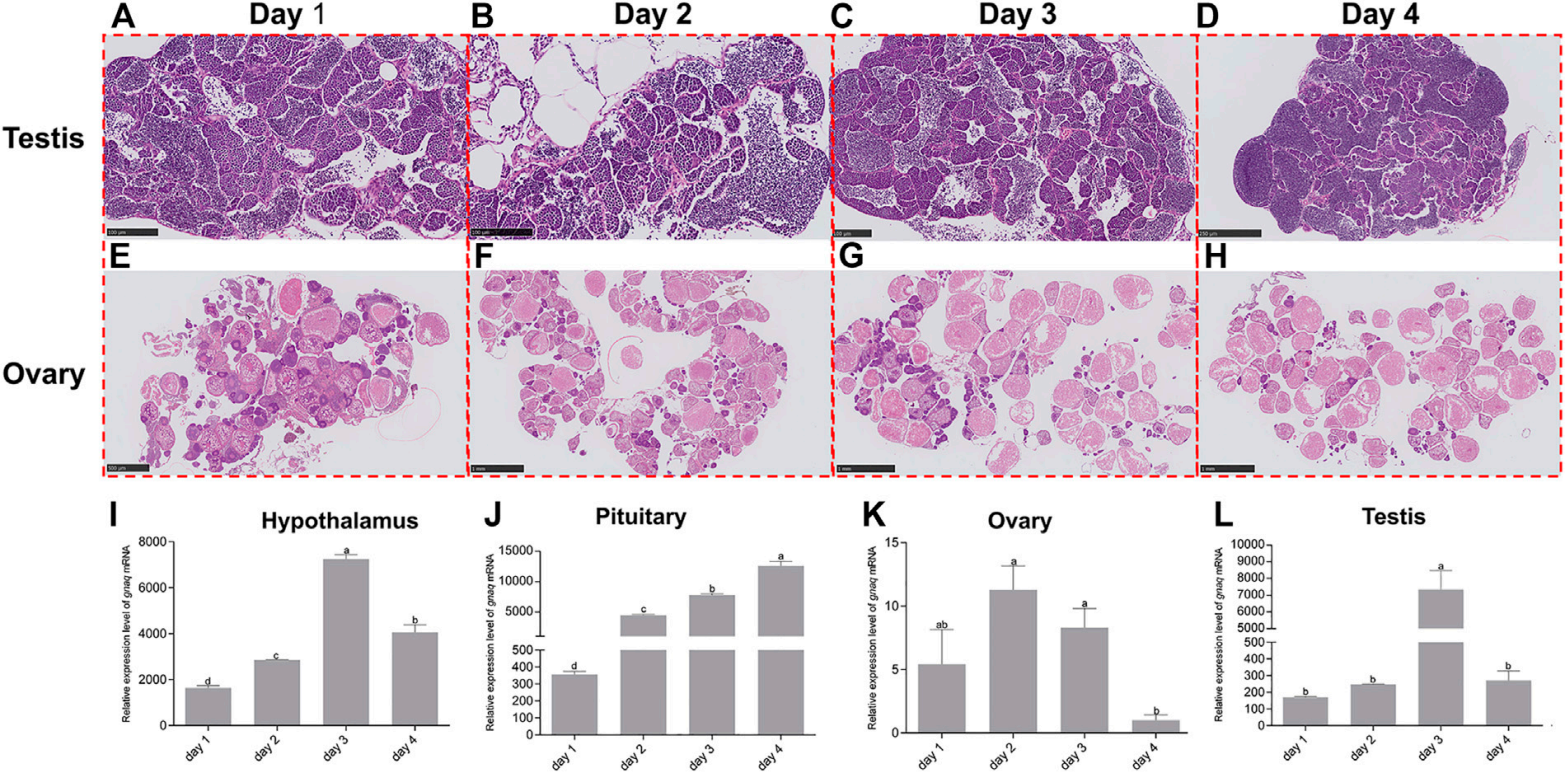
Dynamic changes of *gnaq* expression in HPG axis during the reproductive cycle. **(A)** Testis on Day 1 of the reproduction cycle; scale bar: 100μm; **(B)** testis on Day 2 of the reproduction cycle; scale bar: 100μm; **(C)** testis on Day 3 of the reproduction cycle; scale bar: 100μm; **(D)** testis on Day 4 of the reproduction cycle; scale bar: 250 μm; **(E)** ovary on Day 1 of the reproduction cycle; scale bar: 500 μm; **(F)** ovary on Day 2 of the reproduction cycle; scale bar: 1 nm; **(G)** ovary on Day 3 of the reproduction cycle; scale bar: 1 nm; **(H)** ovary on Day 4 of the reproduction cycle; scale bar: 1 nm; **(I)** expression pattern of *gnaq* mRNA in the hypothalamus during the reproductive cycle; **(J)** expression pattern of *gnaq* mRNA in pituitary during the reproductive cycle; **(K)** expression pattern of *gnaq* mRNA in ovary during the reproductive cycle; **(L)** expression pattern of *gnaq* mRNA in testis during the reproductive cycle. Error bars indicate mean ± SEM (*n* = 5); one-way ANOVA was used for statistical analysis (*p* < 0.05).

In general, zebrafish ovulate every 4–7 days as a reproductive cycle. The zebrafish raised under our conditions ovulated every 4 days; we took 4 days as a reproductive cycle and set the day of ovulation as Day 1. Histological analysis revealed a gradual increase over time in the number of spermatids in the spermatogenic cyst of the testis (Figures 5A–D) and full-growth oocytes in the ovary (Figures 5E–H) after mating, which was consistent with the characteristics of the reproductive cycle.

During the reproductive cycle, the expression of *gnaq* mRNA gradually increased in the hypothalamus, peaking on Day 3 and slightly dropping on Day 4 (the day before the next mating) (Figure 5I). In the pituitary, the *gnaq* mRNA dramatically increased throughout the reproductive cycle and peaked on Day 4, showing a 35-fold increase compared with Day 1 (Figure 5J). In the ovaries, *gnaq* expression was highest on Day 2 and lowest on Day 4 (Figure 5K). As with the testis, the expression of *gnaq* mRNA increased greatly on Day 3—up to 43-fold (Figure 5L).

## Discussion

To date, G-protein signaling has been widely studied in mammals. The Gα subunit structure consists of a GTPase domain and a helical domain. In this work, we identified a member of the G proteins, *gnaq*, from zebrafish; the multiple sequence alignment showed that its GTPase domain and helical domain were strongly conserved among vertebrates (Figure 1A). The phylogenetic analysis also supported the evolutionary conservation of Gαq in vertebrates (Figure 1D). All this evidence implies that fish *gnaq* might have similar functions as in mammals. In the latter, *GNAQ* is highly expressed in the brain (Syrovatkina et al., 2016) and mainly functions in the upstream reproductive development regulation (Ohtaki et al., 2001; Liu et al., 2008; Zhang et al., 2013; Novaira et al., 2014). We thus propose the hypothesis that fish *gnaq* might have an important role in reproduction.

We firstly investigated zebrafish *gnaq* expression patterns in embryos and adults. The consequent *gnaq* expression pattern was consistent with that of maternal factors during embryonic development (Dosch et al., 2004), revealing a potential role of *gnaq* in embryonic development. The WISH and immunohistochemical results confirmed that the *gnaq* mRNA signal was localized in the brain and concentrated in the hypothalamus after this was formed (Figures 3B,D). Meanwhile, the tissue distribution of *gnaq* was partly similar to that of mammals, and *gnaq* was highly expressed in the zebrafish brain, olfactory brain, and hypothalamus (Figure 2). All this evidence indicates that fish *gnaq* probably functions in the nervous system, which is critical in reproductive maturation. In addition, *gnaq* has the highest expression in the olfactory brain and high expression in the spleen, suggesting possible roles for *gnaq* in olfactory development and fish immunology.

In mature zebrafish, GnRH3 is located at the preoptic area-hypothalamus (POA-hypo) and is present in the GnRH3 neurons of olfactory region origin which are prerequisite for normal oocyte development and reproduction (Abraham et al., 2010). Furthermore, α-Melanocyte-stimulating hormone (α-MSH) and agouti-related protein (AgRP)-immunoreactive (ir) cells are found in the ventral zone of the periventricular hypothalamus (Hv) (Forlano and Cone, 2007). Interestingly, Gαq immunohistochemical results in a section of the hypothalamus showed that it was also highly expressed in DIL, Hv, and secretory cells, suggesting that *gnaq* might play a role in various vital activities, including oocyte development, reproduction, the synthesis and secretion of neuropeptides, and energy homeostasis. Although the Gαq location was investigated, its function mechanism is still unknown.

*Gnaq* exhibited the relatively lowest expression in zebrafish gonads, especially in testis (Figure 2), which is very different to mammals: *GNAQ* has a relatively high expression in human gonads (Chen et al., 1996). On the other hand, *gnaq* expression dramatically changed in zebrafish gonads during the reproductive cycle (Figures 5K,L), suggesting that the functional importance of one gene does not depend on how much it is expressed in tissues. The *gnaq* function in fish gonads is still worth study.

Subsequently, an immunohistochemical experiment was conducted to explore the Gαq protein distribution in gonads. Many genes associated with reproduction, such as Dmrt1, Folx2, and Lhr, are detected in gonads (Yung et al., 2014; Lin et al., 2017; Yang et al., 2017). As shown, the Gαq signal was abundant in the oocytes of all stages, especially in immature oocytes (Figure 4A), indicating that Gαq decreased gradually with oocyte development and has a potential role in oocyte development. In the testis, positive signals were observed in spermatogonia, and primary and secondary spermatocytes. Surprisingly, the Gαq signal was not found in spermatids (Figure 4C), suggesting that *gnaq* might function in the early development of testis. In mammals, LHR can also activate Gαq/11 in a dependent manner (Castilho et al., 2014). In knockout of G Gαq/11 in mouse granulosa cells, LHR failed to fully induce the expression of the progesterone receptor (PGR), resulting in follicular rupture defects (Breen et al., 2013). Interestingly, the Gαq signal was similar to LHR in oocytes and testes (Yung et al., 2014; Phoophitphong et al., 2017), suggesting that *gnaq* has a potential function in follicular rupture and testis development—although he nature of this is unclear. On the other hand, *gnaq* was located in secretory cells, as was GnRH (Tsai, et al., 2003). The gonadotropin-releasing hormone (GnRH) is the master regulator of fertility (Tzoupis et al., 2020). In mammals, depletion of GnRH led to defective gonad development and hypogonadism (Pandolfi et al., 2019; Nandankar et al., 2021). Surprisingly, gnrh2 mutant, gnrh3 mutant, and double mutant zebrafish all showed no reproductive defects (there are only two forms of gnrh in zebrafish) (Spicer et al., 2016; Marvel et al., 2018; Marvel et al., 2019). Does *gnaq* have similar results in fish? This needs further investigation using gene-editing technology.

During the reproductive cycle, *gnaq* mRNA in the hypothalamus–pituitary–gonadal axis dramatically changed. Its expression increased gradually in the hypothalamus, peaking on Day 3 (Figure 5I), while *gnaq* dramatically increased in the pituitary throughout the reproductive cycle and peaked on Day 4 (Figure 5J). This suggests that *gnaq* in the hypothalamus and pituitary might function sequentially in time. The *gnaq* expression in the ovary and testis both exhibited the lowest level on Day 4 (Figures 5K,L), at which stage the number of full-growth oocytes or spermatids are in a majority (Figures 5D,H). This explains the low expression of *gnaq* in the mature gonads (Figure 2) and the immunohistochemical results in gonads (Figures 4A,C). Remarkably, the expression of *gnaq* mRNA in the testis increased 43-fold on the third day, indicating that *gnaq* might have a potential role in spermatogenesis (Figure 5L). However, the functional relationship between *gnaq* and reproduction in fish is still not clear.

Taken together, the evolutionary conservation, expression pattern in tissues and embryonic development, and dynamic change during the reproduction cycle suggest that zebrafish *gnaq* is involved in the hypothalamus–pituitary–gonadal axis and has a potential role in the fish reproduction process. The involved molecular mechanism is worthy of further exploration.

## Data Availability

The sequence data have been deposited in NCBI repository, accession number NM_001144799.1.
