# Supplementary material for: Identification and expression analysis of zebrafish gnaq in the hypothalamic–Pituitary–Gonadal axis
Source: Front Genet. 2022 Nov 10;13:1015796. doi: 10.3389/fgene.2022.1015796 (PMC9685404; doi:10.3389/fgene.2022.1015796)
Supplement: Supplementary file 1 [file Table1.DOCX]

**Supplement Table 1**

Tab. 1 primers used in experiment

| primers | sequence (5′-3′) | purpose |
| --- | --- | --- |
| *gnaq*-F | ATGACGCTGGACTCCATCATG | clone |
| *gnaq*-R | TCACACCAGATTGTATTCTTTA |  |
| *gnaq*-qF | TTGTCGACCTGAATCCAGACTC | qPCR |
| *gnaq*-qR | TCTTTGACAGCAGCGAACAC |  |
| β-actin- qF | CTTGCGGTATCCACGAGAC | qPCR |
| β-actin- qR | GCGCCATACAGAGCAGAA |  |
| ef1α-qF | AGGCTGACTGTGCTGTGCTGA | qPCR |
| ef1α-qR | CCAGGGTGAAAGCCAGGAGG |  |
| *gnaq*-tF | ATGACGCTGGACTCCATCAT | WISH |
| *gnaq*-tR | TAATACGACTCACTATAGGGTCACACCAGATTGTATTCTTTAAGCG |  |
