# Supplementary figures and images for: Identification and expression analysis of zebrafish gnaq in the hypothalamic–Pituitary–Gonadal axis
Source: Front Genet. 2022 Nov 10;13:1015796. doi: 10.3389/fgene.2022.1015796 (PMC9685404; doi:10.3389/fgene.2022.1015796)

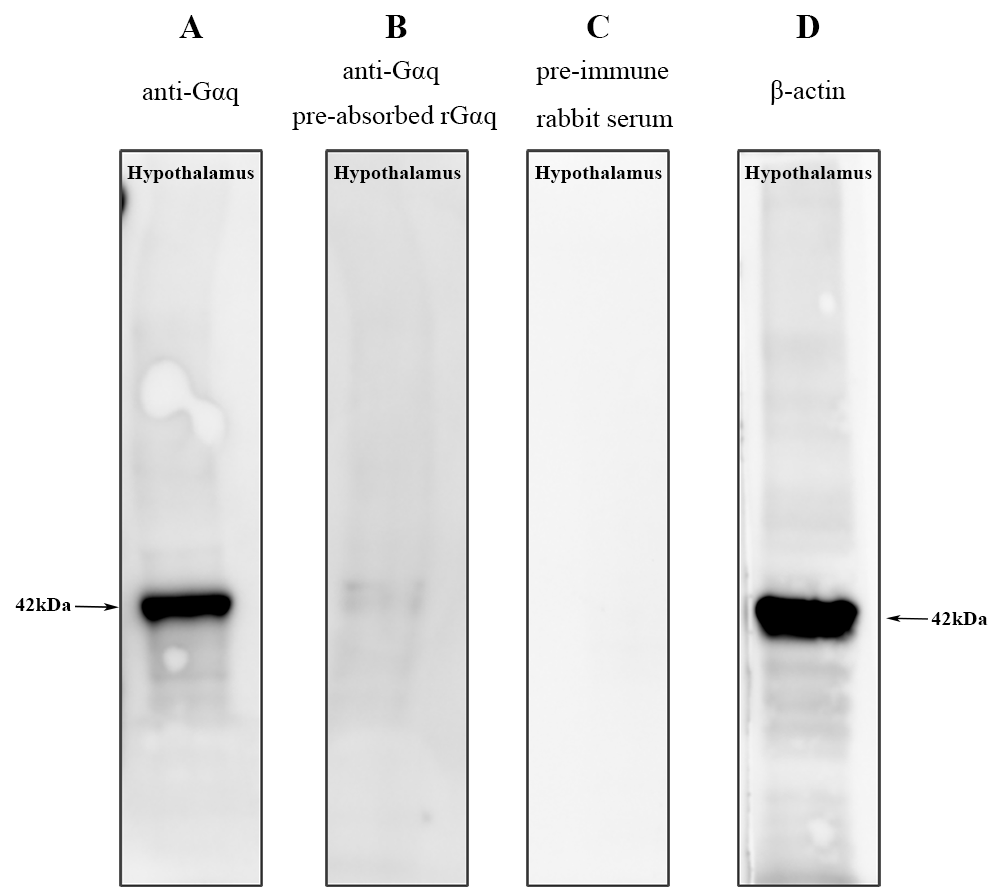

Supplement: Supplementary file 2 [file Image2.TIF]

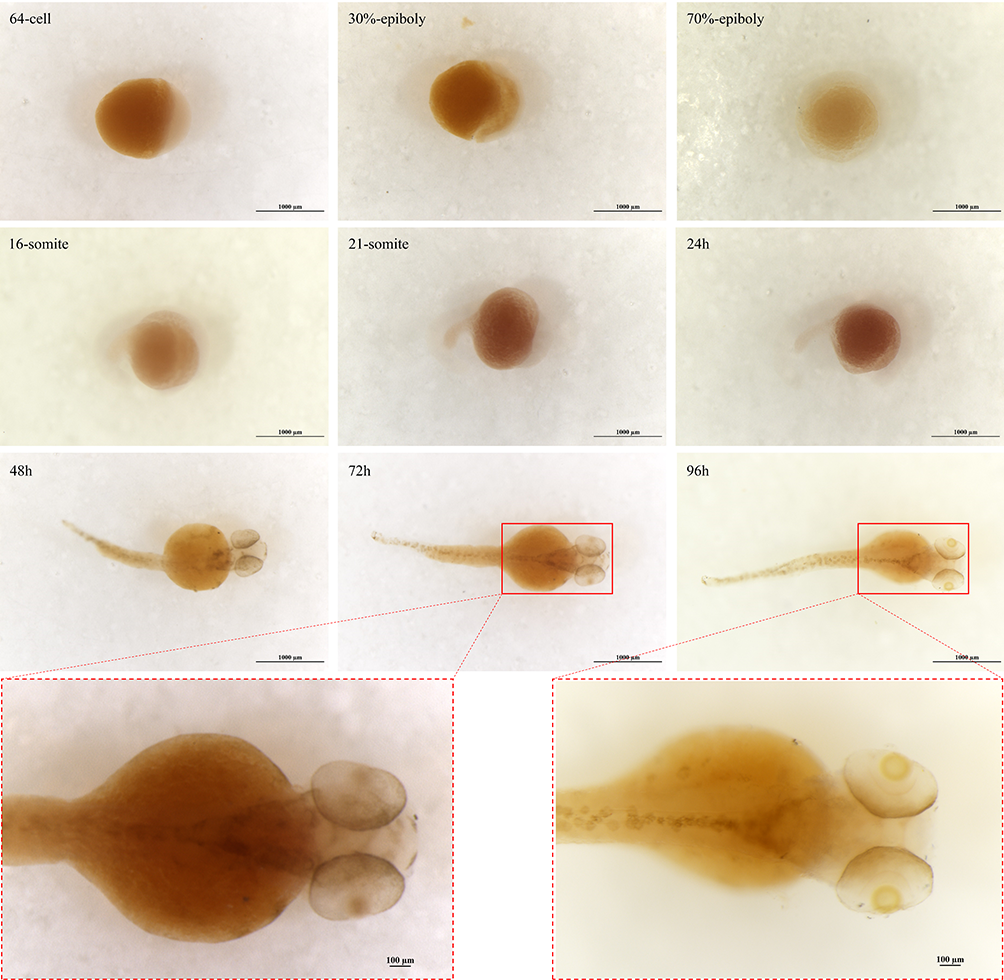

Supplement: Supplementary file 3 [file Image1.TIF]
